# A Literature Review on the Coexisting Chronic Obstructive Pulmonary Disease and Heart Failure

**DOI:** 10.7759/cureus.47895

**Published:** 2023-10-29

**Authors:** Tasniem Tasha, Anjali Desai, Anjana Bajgain, Asna Ali, Chandrani Dutta, Khadija Pasha, Salomi Paul, Muhammad S Abbas, Sondos T Nassar, Lubna Mohammed

**Affiliations:** 1 Internal Medicine, California Institute of Behavioral Neurosciences & Psychology, Fairfield, USA; 2 Psychology, California Institute of Behavioral Neurosciences & Psychology, Fairfield, USA; 3 Family Medicine, California Institute of Behavioral Neurosciences & Psychology, Fairfield, USA; 4 Pediatrics, California Institute of Behavioral Neurosciences & Psychology, Fairfield, USA; 5 Psychiatry, California Institute of Behavioral Neurosciences & Psychology, Fairfield, USA; 6 Medicine and Surgery, California Institute of Behavioral Neurosciences & Psychology, Fairfield, USA

**Keywords:** mortality, management, coexistent disease, heart failure, copd: chronic obstructive pulmonary disease

## Abstract

The convergence of chronic obstructive pulmonary disease (COPD) and heart failure (HF) is a prevalent yet often overlooked medical scenario. This coexistence poses diagnostic challenges due to symptom similarities. This comprehensive review extensively examines the impact of COPD and HF on pharmacological management. Furthermore, the concurrent presence of these conditions amplifies both mortality rates and societal financial strain. Addressing these intertwined ailments necessitates a multidisciplinary approach. Within this review, we delve into the foundational mechanisms, diagnostic intricacies, and available management choices for these closely related conditions.

## Introduction and background

Chronic obstructive pulmonary disease (COPD) and heart failure (HF) are the two commonly coexisting disorders. It can be difficult to distinguish the relative contributions of these diseases because symptoms sometimes overlap. COPD, when coexistent with heart failure, may significantly impact its prognosis, or vice versa. The influence of one can affect the disease progression, clinical outcome, and mortality of the other. It can be either through common risk factors, one disease enhancing the risk or compounding the severity of another, or both. Understanding the link between COPD and heart failure has significant implications for disease management, such as focused therapies and medication warnings. In observational studies and randomized controlled trials (RCTs), the prevalence of COPD among patients with HF ranges from 10% to 20% [1].

The global initiative for COPD states that significant exposure to noxious particles or gases is typically the primary cause of persistent respiratory symptoms and airflow limitation brought on by airway and/or alveolar abnormalities. COPD is a leading global cause of illness and mortality and a significant economic burden. COPD is frequently placed among the top causes of mortality in the United States, killing over 120,000 people each year [2,3]. Prior to the Coronavirus Disease 2019 (COVID-19) pandemic, it was the world's third-leading cause of death [4].

On the other hand, heart failure (HF) is a complex syndrome characterized by impaired ventricular ejection of blood or abnormal left ventricular (LV) and/or right ventricular (RV) filling, as well as elevated filling pressure [5]. Simply put, HF may result from any structural or functional cardiac condition that compromises the ventricle's capacity to receive or expel blood. HF and COPD coexist, with the frequency of COPD ranging from 10% to 20% in randomized controlled trials (RCTs) and observational studies [1].

COPD overlaps certain basic symptoms like dyspnea, chest discomfort, and fatigue with heart failure. COPD, in turn, may be linked to worsened heart failure outcomes [6,7]. It's unclear exactly how COPD and cardiovascular problems are related. However, there is evidence that COPD and cardiovascular disorders like heart failure are associated with low-grade systemic inflammation [8]. Patients have a higher risk of underlying ischemic heart disease with moderate or severe airflow obstruction and high circulating C-reactive protein (CRP), which indicates that this inflammatory marker has an effect on cardiac risk. Although not consistent, patients with COPD show a link between CRP and vascular structure and activity. Inflammation has been linked to the pathogenesis of HF. Despite the presence of known risk factors, this condition was more prevalent in Framingham subjects with elevated CRP and cytokine levels [9].

**Figure 1:**
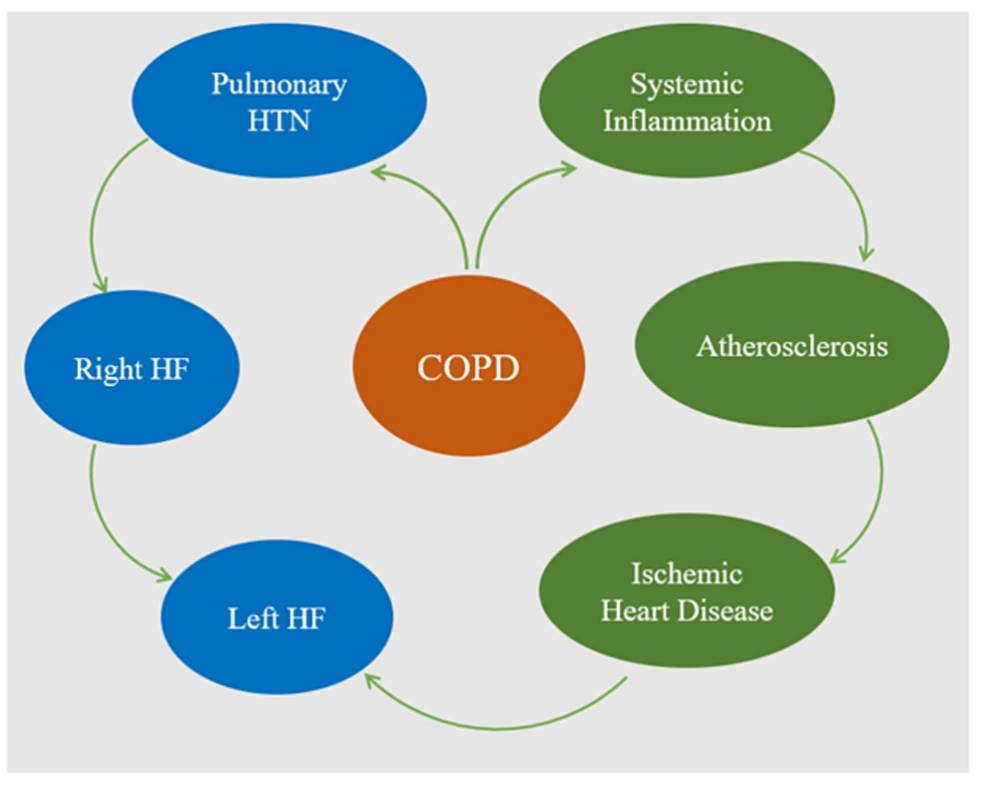
Schematic presentation of the underlying pathophysiology of coexisting COPD and heart failure HTN: hypertension; HF: heart failure; COPD: chronic obstructive pulmonary disease

One theory that explains the high prevalence of left ventricular systolic dysfunction in COPD patients is that systemic inflammation accelerates coronary atherosclerosis, leading to the development of ischemic heart disease. The association between these chronic progressive diseases may possibly be explained by the left ventricular dysfunction that we find in COPD patients [10]. Conversely, severe COPD often causes pulmonary hypertension, which might cause right HF. Furthermore, right heart failure can lead to left HF in the long run [11]. Furthermore, regardless of lung function impairment, nearly half of COPD patients have a coexisting metabolic syndrome and elevated levels of systemic inflammatory markers [12,13]. Diabetes is also linked to decreased lung function, and obesity may also make ventilatory mechanics even worse [14]. The major risk factors for cardiovascular disease are diabetes, physical inactivity, and metabolic syndrome, along with their individual components. The fact that both act via pro-inflammatory mechanisms adds weight to the argument that low-grade systemic inflammation is a common pathophysiological connection between COPD and cardiovascular disease [15]. In Figure 1, we have presented a schematic presentation of the underlying pathophysiology of coexisting COPD and heart failure.

Additionally, studies have demonstrated that the potential side effects of COPD medications may result in cardiovascular complications, while those used for HF management may worsen COPD symptoms [16,17]. This is observed when cardiovascular disorders are treated with beta-blockers (BB) and respiratory disorders with beta-2 agonists [18,19]. Smoking also increases the risk of developing COPD and heart failure. In the following sections, we have attempted to reflect on the influence of the coexistence of COPD and heart failure on diagnosis, treatment, and prognosis.

## Review

Method

**Figure 2:**
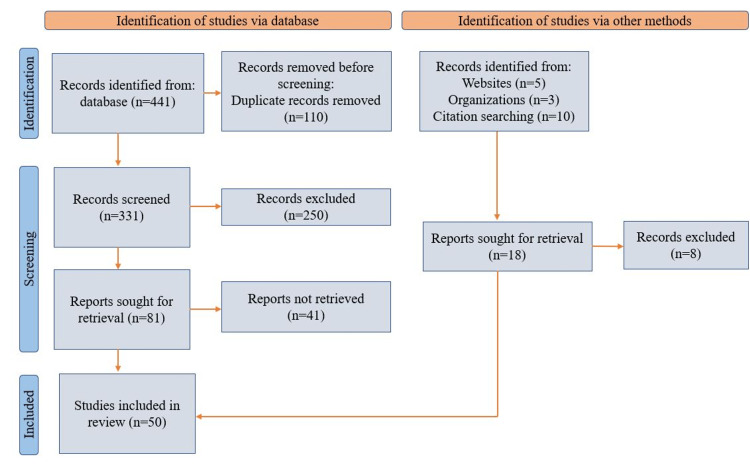
Study selection process for this literature review

We employed the search strategy using PubMed and Google Scholar electronic databases, covering the period between 2017 and 2022. The keywords we used were “heart failure,", “COPD”, “coexistence,” and "management," which yielded a large number of articles. These keywords were combined to narrow down our search. The Medical Subject Headings (MeSH) words “heart failure”, “COPD”, and “coexistence” were also used to jot down the articles, further selecting those with free full texts, abstracts in the English language, and on humans only. After the articles were assessed and ineligible articles were ruled out, a total of 50 articles were selected to serve as evidence for our literature review. The study selection process inspired by Preferred Reporting Items for Systematic Review and the Meta-Analysis (PRISMA) 2020 flow diagram [20] for this review is shown in Figure 2.

Diagnosis

Both COPD and HF share similar signs and symptoms. Both disorders frequently exhibit signs of fatigue and exertional dyspnea, which can cause pronounced activity intolerance [21]. However, without the presence of infectious aggravation of COPD, nocturnal cough, paroxysmal nocturnal dyspnea, ease of fatigue, and impaired exercise tolerance lead to a diagnosis of HF. In COPD, the prevalence of right ventricular failure should be inferred from the presence of jugular venous distension, ankle edema, and hepatomegaly [22]. When the systolic ventricular function is normal, the negative predictive value is reported as high in the electrocardiogram for diagnosing HF. However, with the presence of abnormalities, it is not a specific method to diagnose HF. These abnormalities are quite common in patients who have both COPD and HF [23].

Furthermore, chest hyperinflation reduces the cardiothoracic ratio, making the heart appear long and narrow. Because of radiolucent lung fields and pulmonary vascular remodeling, pulmonary edema is also hidden. Chest radiography is not promising for detecting HF or COPD; however, it may be beneficial for detecting other diseases [21].

In patients with COPD, plasma levels of natriuretic peptides are a quick and sensitive indicator of HF. Both B-type natriuretic peptide (BNP) and the N-terminal fragment of B-type natriuretic peptide (NT-proBNP) are derived from proBNP, a prohormone generated by myocytes to increase atrial and ventricular filling pressure [24]. A single BNP cutoff value of 100 pg/mL is used to exclude or identify HF. If BNP levels are reported below the cutoff value, HF is rare. BNP values in COPD patients ranging from 100 pg/mL to 500 pg/mL may be attributed to cor pulmonale (right ventricular strain), mild left ventricular failure, or both. Finally, although not explicitly studied in COPD patients, the BNP value of 500 pg/mL suggests severe HF in patients with COPD, which multiple authors propose [8,21,25]. National Institute for Health and Care Excellence (NICE) guidelines recommend that an individual with BNP levels > 400 pg/mL or NT-proBNP > 2000 pg/mL should have echocardiography within two weeks, or within six weeks for patients if BNP values lie between 100 pg/mL and 400 pg/mL or NT-proBNP between 400 pg/mL and 2000 pg/mL [26]. Techniques for cardiac imaging might be applied if there are any doubts. In COPD patients, echocardiography can identify left ventricular dysfunction (diastolic or systolic), which is frequently linked to the existence of cardiovascular illness [27]. Additionally, to assess whether corpulmonale is present in COPD patients and to identify their short-term prognosis, the right ventricle must undergo an echocardiographic evaluation, which measures the systolic pulmonary artery and interventricular septal pressures [28]. HF is eliminated if echocardiography is normal. However, patients with COPD should be evaluated for HF diagnosis who have abnormal left ventricular mass, an enlarged left atrium, and a left ventricular ejection fraction > 40% [29].

Despite being the gold standard for diagnosing HF, echocardiography may be limited to individuals with obesity or COPD who have a poor echocardiographic window because of pulmonary hyperinflation [30]. Using magnetic resonance imaging (MRI) to assess the right ventricle in certain situations may be more effective. Emphysema and COPD patients have obstructive patterns, whereas heart failure patients have restrictive ones. As a result, when a patient has both COPD and HF, the pulmonary function test can show both obstructive and restrictive configurations [31].

**Figure 3:**
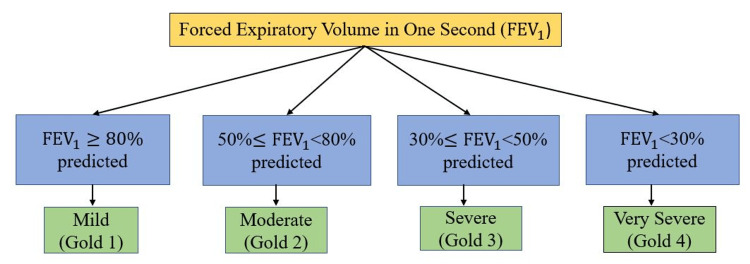
Global Initiative for Chronic Obstructive Lung Disease (GOLD) classification of airflow limitation severity in chronic obstructive pulmonary disease (COPD) (in patients with FEV1/FVC < .70) FEV1: forced expiratory volume in one second; FVC: forced vital capacity; Gold: Global Initiative for Chronic Obstructive Lung Disease; COPD: chronic obstructive pulmonary disease

Figure 3 reports the Global Initiative for Chronic Obstructive Lung Disease (GOLD) criteria [32]. According to GOLD criteria, the post-dilatory ratio of forced expiratory volume in one second (FEV1) and forced vital capacity (FVC) must be less than 0.7 (FEV1/FVC < 0.7) when using spirometry to diagnose COPD. Lung function can eventually improve in HF patients with the proper care. After HF treatment, it is suggested to repeat the spirometry test to determine the patient's ultimate COPD status [33].

Pharmacological treatment of heart failure in patients with chronic obstructive pulmonary disease

It is not evident to suggest that HF should be treated differently in the presence of COPD. Thus, patients with this respiratory condition should receive treatment for HF following standard HF guidelines [34]. The symptoms and survival rate of patients with chronic HF can be improved by beta-blocker therapy. However, it is generally avoided in patients with COPD due to worries about the beta-2-agonist bronchodilator effect and the worsening of bronchospasm [35]. There is no evidence that (cardioselective) beta-blocker therapy reduces the respiratory benefits or increases the cardiovascular risk of inhaled long-acting beta-agonists in terms of overall survival or frequency of COPD exacerbations [36,37], and it has some cardiovascular and mortality benefits [38]. On the other hand, nonselective beta-blockade can cause bronchospasm in those who are predisposed, but selective beta-1 blockers (e.g., atenolol or metoprolol) are proven safe in COPD patients, even in the presence of bronchospastic components [38].

The morbidity and mortality of COPD patients can be decreased with treatment with statins, angiotensin-converting enzyme (ACE) inhibitors, and angiotensin receptor blockers (ARBs) [39,40]. Some retrospective investigations, for example, by Mancini et al. [39], have demonstrated a decreased risk of hospitalization in statin-treated COPD patients who also received treatment with ACE inhibitors and/or ARBs. They also suggest that the combination of ACE inhibitors and ARBs could be a treatment option for increasing survival in patients with absolute contraindications for blockers [39]. Furthermore, Mortensen et al. [40] reported that using statins and ACE inhibitors before hospitalization is associated with lower mortality in subjects hospitalized for COPD exacerbations.

Another important factor to consider in these patients is the use of diuretics, as high doses of loop diuretics can cause metabolic alkalosis, with hypoventilation as a compensatory mechanism, which can worsen hypercapnia [41]. Table 1 summarizes the pharmacological management of patients with HF and COPD in stable and unstable conditions [42].

**Table 1:** Management of patients with HF and COPD in stable and unstable conditions HF: heart failure; COPD: chronic obstructive pulmonary disease; RAAS: renin-angiotensin-aldosterone system; ACEi: angiotensin-converting enzyme inhibitor; ARB: angiotensin receptor blocker; LABA: long-acting beta2 agonist; LAMA: long-acting muscarinic agonist; NIV: non-invasive ventilation; ACPE: acute cardiogenic pulmonary edema; AECOPD: acute exacerbation of chronic obstructive pulmonary disease

Condition	Disease	Management
Stable	HF	Diuretics, Beta-blocker, RAAS inhibitors (ACEi, ARB)
	COPD	LAMA and/or LABA, Inhaled corticosteroid
Unstable	HF	Diuretics, Beta-blocker, RAAS inhibitors (ACEi, ARB), NIV (ACPE)
	COPD	Bronchodilators (Beta 2 agonist), Systemic corticosteroids, Antibiotics, NIV(AECOPD)

Pharmacological treatment of chronic obstructive pulmonary disease in patients with heart failure

According to clinical guidelines, COPD should be treated in patients with HF because there is no direct evidence that this respiratory disease should be treated differently in the presence of HF [34]. This statement is based on long-term study findings in patients with HF and comorbid COPD [43-45]. Some studies, but not all, have suggested that beta-2 agonists may be detrimental to patients with left ventricular dysfunction. In a study of 1529 patients with left ventricular systolic dysfunction (as determined by echocardiography or radionuclide ventriculography), inhaled beta-agonists were associated with a dose-response relationship in terms of hospitalization for heart failure [46]. In contrast, after controlling for age, gender, smoking, medications, and severity of comorbidities, a retrospective study of 1294 subjects enrolled in a heart failure disease management program found no increase in mortality associated with beta-2 agonist use (hazard ratio 1.043, 95% confidence interval 0.771 to 1.412) [47].

Mechanical ventilation provided through a noninvasive interface (e.g., a face mask, nasal mask, orofacial mask, or nasal prongs) is referred to as non-invasive ventilation (NIV), which is also known as noninvasive positive pressure ventilation (NPPV). NIV lowers mortality and intubation rates and is the recommended technique of ventilatory support in many COPD exacerbations [48]. A group of patients with acute respiratory acidosis (partial pressure of carbon dioxide (PaCO_2_)>45 mmHg or pH 7.35) are expected to benefit from an initial trial of NIV with bilevel-positive airway pressure. A trial of NIV is also appropriate for other individuals with non-hypercapnic respiratory failure related to COPD exacerbations; however, the derived benefit may be considerably smaller.

Bilevel NIV improves hypercapnic respiratory failure in acute exacerbation of chronic obstructive pulmonary disease (AECOPD) by improving alveolar ventilation, as evidenced by improved respiratory mechanics (e.g., decreased respiratory rate, increased tidal volume, and increased minute ventilation) and gas exchange parameters (e.g., increase in partial pressure of Oxygen (PaO_2_) and decrease in PaCO_2_) [49]. Preload reduction, avoidance of alveolar collapse at end expiration, and decreased left ventricular afterload are hypothesized to be the mechanisms through which NIV reduces acute cardiogenic pulmonary edema (ACPE). The literature proposes a trial of NIV, usually in conjunction with continuous positive airway pressure (CPAP), for patients with ACPE. NIV reduces the need for intubation, improves clinical and laboratory indicators of respiratory failure (e.g., heart rate, dyspnea, hypercapnia, acidosis), and lowers mortality in patients with ACPE, according to meta-analyses of small, randomized studies [50]. In summary, NIV improves the prognosis of patients with acute respiratory failure due to hypercapnic exacerbations of COPD or heart failure in situations of acute pulmonary edema when combined with conventional treatment.

## Conclusions

Heart failure and COPD frequently coexist, yet this fact is frequently overlooked. COPD is widely acknowledged to have a negative impact on the prognosis of heart failure, though more conclusive research is needed. To reduce symptoms, delay progression, and improve prognosis, diagnosing these two coexisting conditions is imperative, as is establishing a management strategy that simultaneously addresses both comorbidities. This is especially true given the rising mortality rate and the significant negative effects each disease has on quality of life and performance status.
